# Higher Medical Education in the Southern States

**Published:** 1892-11

**Authors:** 


### HIGHER MEDICAL EDUCATION IN THE SOUTHERN STATES.

The circular recently sent out from Nashville, with the names of Drs. W. T. Briggs and W. D. Haggard, invites the medical schools of the South to a convention in Louisville, Ky., during the session of the Southern Surgical and Gynecological Association, which will be held on the 15th, 16th, and 17th of November, next.

The object of this conference is to adopt a uniform standard of requirements by all the Southern schools, and thus place them on an equal basis before the students who may seek a place for pursuing their studies. Without coöperation in the requirements for graduation, it is evident that nothing can be accomplished in advancing the cause of medical education, either by lengthening the term, defining the preliminary qualifications, or exacting a thorough knowledge of the different branches for receiving the degree of M. D. Should the schools of one State require a three years' course of study, while others decline to do so, the most probable result would be, that students who are anxious to get a degree by the shortest route, without reference to a high order of professional attainments, would patronize the schools having a two years' course. Had the proposed bill before the Legislature of Georgia become a law, and exacted from the schools of this State a requirement of three years' attendance on the part of the students, it must have proven suicidal to the Georgia medical colleges, while colleges of other Southern States continued to graduate students with only two years' attendance. The example of the medical college of South Carolina, at Charleston, in establishing a three years' course, serves to illustrate this point in a most signal manner. If the great falling off in numbers, which has followed this premature step, does not suffice to convince those in authority in Georgia of the fate which must have attended a requirement of three years in advance of the other schools in the South, no reasoning can avail to satisfy them.

However desirable it may be to raise the standard of medical education, the question of expediency in undertaking to advance the requirements without coöperation, stares us in the face ; and if all can be induced to move together by such action as is proposed in this convention, the interest of all concerned will be promoted.

The Southern Medical College has signified its acceptance of the invitation to join the convention at Louisville, and it is to be hoped that every Southern school may accede to the proposition to adopt a three years' course and insist upon the proper preparation for entering upon the study of medicine.

Some definite understanding should also be reached in regard to the qualifications for graduation, so that a wholesale granting of diplomas shall discredit the school which tacitly, if not publicly, gives all applicants to understand that they will receive their degree.

It is not enough that the graduates of such a school shall fail to pass the examination of the medical boards in a neighboring State, however humiliating this may be to the poorly trained victim, but the school discharging such material upon the suffering people of this country should be held to accountability.

The above is part of an editorial which appears in the *Southern Medical Record*, October, 1892.

Believing the writer, Dr. J. McF. G., to be on the right line, and that his suggestions are sound and at the same time practical, we have reproduced what he has said. There must, unquestionably, be coöperation on the part of all the leading Southern medical colleges before any good results can be attained. Alabama is ready.—*Medical and Surgical Age.*

